# Development of a Portable SPR Sensor for Nucleic Acid Detection

**DOI:** 10.3390/mi11050526

**Published:** 2020-05-21

**Authors:** Yafeng Huang, Lulu Zhang, Hao Zhang, Yichen Li, Luyao Liu, Yuanyuan Chen, Xianbo Qiu, Duli Yu

**Affiliations:** 1 College of Information Science and Technology, Beijing University of Chemical Technology, Beijing 100029, China; 15538975363@163.com (Y.H.); zh1632414919@163.com (H.Z.); yichenli_1918@163.com (Y.L.); luyaolukeliu@126.com (L.L.); dyu@mail.buct.edu.cn (D.Y.); 2 Core Facility for Protein Research, Institute of Biophysics, Chinese Academy of Sciences, Beijing 100101, China; chenyy@ibp.ac.cn

**Keywords:** surface plasmon resonance (SPR), portability, angle modulation, nucleic acid detection, hybridization

## Abstract

Nucleic acid detection is of great significance in clinical diagnosis, environmental monitoring and food safety. Compared with the traditional nucleic acid amplification detection method, surface plasmon resonance (SPR) sensing technology has the advantages of being label-free, having simple operation, and providing real-time detection. However, the angle scanning system in many SPR angle modulation detection applications usually requires a high-resolution stepper motor and complex mechanical structure to adjust the angle. In this paper, a portable multi-angle scanning SPR sensor was designed. The sensor only uses one stepping motor to rotate a belt, and the belt pulls the mechanical linkages of incident light and reflected light to move in opposite directions for achieving the SPR angle scanning mode that keeps the incident angle and reflected angle equal. The sensor has an angle scanning accuracy of 0.002°, response sensitivity of 3.72 × 10^−6^ RIU (refractive index unit), and an angle scanning range of 30°–74°. The overall size of the system is only 480 mm × 150 mm × 180 mm. The portable SPR sensor was used to detect nucleic acid hybridization on a gold film chip modified with bovine serum albumin (BSA). The result revealed that the sensor had high sensitivity and fast response, and could successfully accomplish the hybridization detection of target DNA solution of 0.01 μmol/mL.

## 1. Introduction

Nucleic acid is the basic genetic material of creatures and plays an extremely significant role in the growth, reproduction and inheritance of creatures [1,2]. Nucleic acid detection is of great significance and demand in clinical diagnosis, environmental monitoring and food safety [3,4,5]. Currently, including polymerase chain reaction (PCR) and other isothermal amplification technologies, DNA microarray technologies are conventional methods for nucleic acid detection [6,7,8]. The detection method based on PCR is time-consuming and complex to operate. In addition, this method cannot be used for real-time detection [9]. DNA microarray technology is stringent for technical personnel. It requires expensive instruments and complex preparation of samples. Droplet digital PCR is a new method developed on the basis of conventional PCR. It has the advantages of absolute quantification and high sensitivity. Its lower detection limit is lower than that of conventional PCR technology. However, droplet digital PCR relies on expensive instruments and costs a lot, and so its popularity is not high at present [10]. Compared with the mentioned techniques, nucleic acid detection based on surface plasmon resonance (SPR) sensing technology is a label-free, real-time, simple to operate, and highly sensitive detection method [11,12,13,14,15]. SPR sensing technology has been already used to study nucleic acid interactions.

SPR, as a physical optical phenomenon occurring between the metal film and thinner medium on its surface, is very sensitive to the change of refractive index of the media close to the surface of the metal film [16,17]. The position of the resonance absorption peak changes with the refractive index of the medium, which is an inherent feature of all materials. By using this principle, the corresponding material concentration and dynamic characteristics can be detected [18]. Compared with traditional biochemical analysis technology, SPR sensors have been rapidly developed due to their excellent characteristics of real-time monitoring, label-free, less sample consumption and highly sensitive quantitative detection [19]. At present, SPR sensing technology has become a mature method for detection of biomolecular interactions. It has been applied in many fields, such as clinical disease diagnosis, environmental monitoring and quantitative detection of transgenic ingredients, and so on [20]. SPR sensing technology can detect small changes in the refractive index effectively with high sensitivity. However, in order to detect even smaller changes in the refractive index, amplification markers such as liposomes, latex particles and proteins need to be used for further sensitivity improvement [21,22].

In addition to biological methods to improve the detection sensitivity, different optical detection modes of SPR will also affect the sensitivity. The methods of SPR sensing technology mainly include angle modulation, wavelength modulation, phase modulation and intensity modulation [23]. Among them, SPR angle modulation method can obtain a large refractive index measurement range, and its measurement sensitivity is very high, up to 1 × 10^−7^ RIU [24]. However, angle modulation usually requires a high-resolution stepper motor and mechanical to adjust the angle. The complex structure and large volume of this instrument limited the development of point-of-care testing (POCT).

Currently, most commercial SPR instruments which adopt Kretchman prism-coupled optical structure are expensive to purchase and maintain, large in size, complex in structure, and professional services in need [25]. It brings great challenge to the popularization and practical application of SPR instrument. Therefore, miniaturized and portable SPR instruments is the development tendency. Pan et al. developed a portable SPR instrument which has a size of 550 mm × 200 mm × 330 mm, an angle scanning accuracy of 0.002°, a refractive index resolution of 10^−6^ RIU, and an angle scanning range of 64°–84°. It has been successfully applied to the detection of hemocyanin and microcystin in shrimp [26]. Laksono et al. developed a low-cost, high-precision SPR sensing system that applied anti-backlash gear to angle modulation. This kind of anti-backlash gear structure improves the system’s angle scanning range to 30°–70°, and the accuracy is 0.01° [27]. There are also studies using 3D printer technology to manufacture SPR parts to reduce costs. For example, the mechanical alignment components in the SPR instrument developed by Manjunath were made using 3D printing technology. Manjunath developed low-cost spectrometers using diffraction gratings and webcams. He built a dual-channel SPR system with a sensitivity of 10^−4^ RIU using two plastic fibers [28]. Zhang et al. used 3D printing technology of molten bulk molding to make some mechanical parts required by the system to reduce the cost and shorten the development cycle. The angle scanning range of the SPR system can reach 40°–72°, and the resolution measured by the system is 8.34 × 10^−6^ RIU [29]. There are also studies that have combined the versatility of smart electronics with the sensing capabilities of SPR technology. For example, Vestri used the Raspberry Pi single board computer equipped with touch screen to realize the management of the entire measurement process of SPR. Vestri used the CCD image sensor as the signal acquisition, so that the refractive index resolution could reach 4.9 × 10^−6^ RIU [30]. In the above studies, some angle scanning structures were complex, requiring the use of two stepper motors to complete a wide range of angle scanning. Some could not achieve angle scanning. None of these studies achieved an effective combination of high precision, wide range of angle scanning and high detection sensitivity. Therefore, it is necessary to develop an SPR sensor with high precision, wide angle scanning range and high sensitivity.

There have been many studies on SPR sensors for nucleic acid detection. Kim et al. employed a SPR method for the quantitative detection of avian influenza DNA hybridization. The sensor has a refractive index resolution of 1.6 × 10^−4^ RIU. The incident angle of the sensor is fixed at 60°. They used wavelength modulation of the sensor to detect target DNA of 1 μM. The incident angle of this sensor is fixed so that it cannot achieve angle modulation [31]. Ding et al. completed highly sensitive detection of microRNA using SPR biosensors. The target miRNA was first captured by the DNA probes on the surface of the chip, and then streptavidin was employed for signal amplification via binding with biotin on the long DNA super-sandwich assemblies. This signal amplification method made the sensitivity of the SPR biosensor very high. The biosensors could detect target miRNA as low as 9 pM in 30 min using the method [32]. Bagi et al. implemented morpholino-based nucleic acid sensing on a portable SPR instrument. They tested and evaluated two signal amplification methods. A significantly higher response was observed with the sequential approach (up to 16-fold). The binding of the target oligonucleotide labeled with the biotin probe occurred on the sensor surface, and then the streptavidin magnetic beads were bound to the immobilized target. This method could detect a 45-nucleotide long oligo target as low as 0.5 nM (7.2 ng/mL) [33]. Therefore, the methods with high sensitivity for nucleic acid detection adopt the method of biological signal amplification, while for the direct method, the limits of detections are in the nanomolar range, which are weaker than sensitivity-enhancing methods [34]. However, the process of direct detection is simple and convenient for real-time and fast detection in field use.

In this paper, a portable multi-angle scanning SPR sensor was designed. The angle modulation of the sensor uses the mechanical scanning method. It only uses one stepping motor to rotate and drive a belt. The belt pulls the mechanical linkages of incident light and reflected light to adjust the length and angle of the linkages at the same time. The intermediate prism is fixed so that the incident angle is always equal to the reflected angle. The angle scanning accuracy of the sensor is measured to be up to 0.002° and the resolution of the refractive index to be up to 3.72 × 10^−6^ RIU. The sensor can control the angle of incident light and reflected light by the motor to achieve real-time monitoring. Finally, the nucleic acid hybridization experiment was performed on a gold film chip modified with BSA. The experimental result showed that the instrument had good performance and could achieve nucleic acid hybridization detection effectively.

## 2. Materials and Methods

### 2.1. Surface Plasmon Resonance (SPR) Sensor Setup

**Figure 1:**
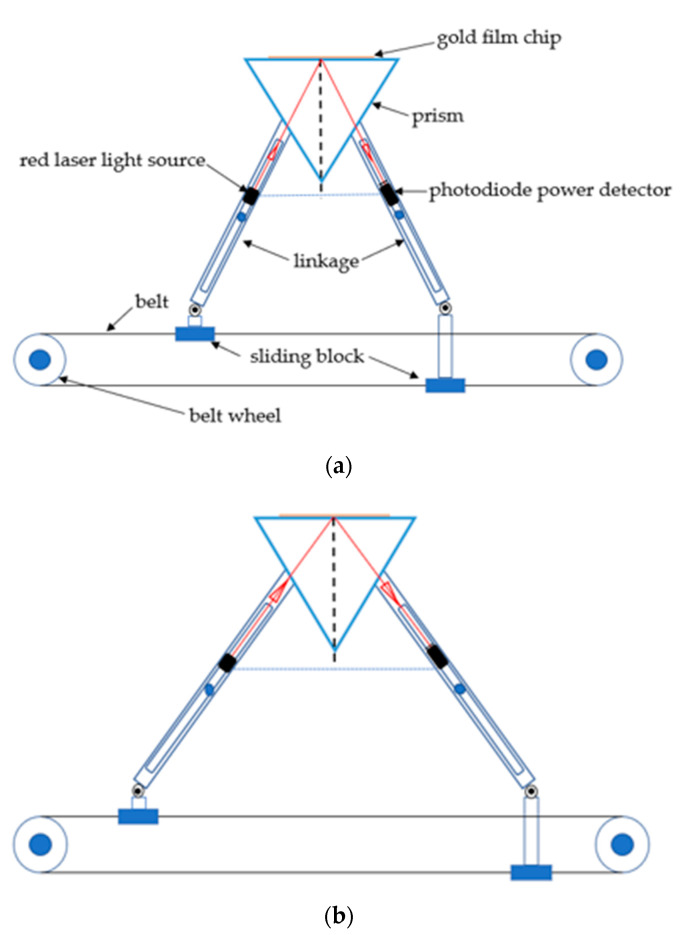
(**a**) Schematic of angle modulation (Position 1); (**b**) schematic of angle modulation (Position 2).

The portable multi-angle scanning SPR sensor includes: a horizontal drive module, symmetrical optics module, and prism and flow cell module. As the motion system of SPR sensor, the horizontal drive module includes a motor, belt wheel, belt, sliding block and guide rail. The two sliding blocks are respectively fixed on the upper surface and the lower surface of the belt. The sliding blocks can generate symmetrical motion along the guide rail under the belt drive. The two sliding blocks drive two linkages on each side of the incident and reflected light. One end of the linkages on each side is respectively fixed on the left or right sliding blocks and the other end is fixed on an axis of rotation as the center of rotation. The two linkages on each side are connected in parallel. The total length of the two linkages can be adjusted automatically so that the linkages of the left and right side produce symmetrical motion. The principle of angle modulation is presented in Figure 1.

The portable multi-angle scanning SPR sensor only requires a stepper motor to drive the belt. The sliding block on the belt is used to pull the incident light linkages and reflected light linkages on the symmetrical optical module to move symmetrically. The prism position is fixed. This structure makes the incident angle equal to the reflected angle at all times. If the prism position is adjusted up and down, the angle detection range can be expanded. The incident angle of the sensor can change from 30° to 74°. The detection from the gas phase to liquid phase can be performed. The red laser light source (wavelength is 628 nm) is fixed on the incident light linkages. A standard photodiode power detector is installed on reflected light linkages (Note: fix the detector in a position where the light can be concentrated in the center of the photosensitive area). The photoelectric signal collected by the photodiode power detector (Thorlabs) is processed by the digital signal processing chip and then the signal is transmitted to the computer. The computer can display the relation graph of light intensity and time in real time. The SPR system can be operated by a laptop for field applications in this paper. The prism and flow cell module uses the classic Kretschmann prism structure. This module uses a triple prism (n = 1.72; Nanjing, China).

**Figure 2:**
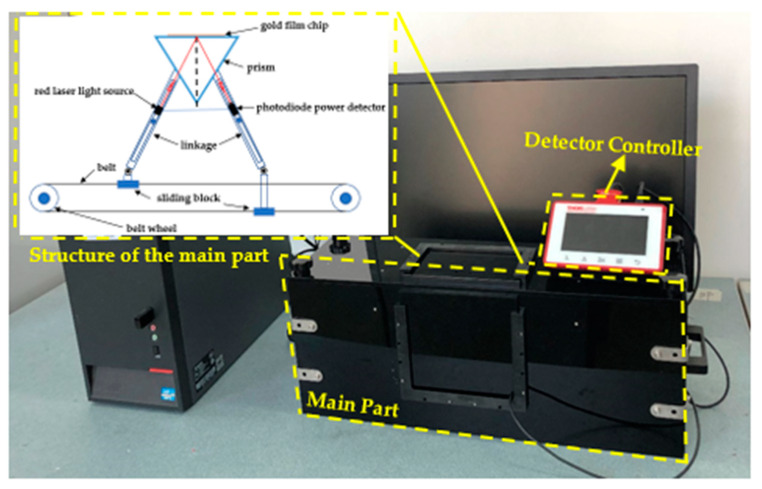
Real surface plasmon resonance (SPR) instrument set-up in this paper.

In practice, a glass slide sputtered with metal (usually gold) was attached to the top surface of a prism with matching oil. Then, the flow cell was balanced on the glass slide through the pressure structure. In order to prevent the interference of natural light, the sensor was designed with a light-proof structure. The overall size of the SPR sensor is 480 mm × 150 mm × 180 mm, and the total weight is 3.09 kg. The prototype SPR instrument is presented in Figure 2.

### 2.2. SPR Chip and the Flow Cell

Two kinds of SPR sensor chip were used in this paper. The first chip was fabricated by magnetron sputtering technology on 20 mm × 20 mm × 0.17 mm glass slide. In this process, 2 nm of chromium and 50 nm of gold was sputtered in an area of 5 mm× 5 mm successively. Before the experiment, the metal surface was cleaned with a sterile cotton soaked in alcohol and then was dried. The second chip was made by modifying BSA on the first chip. The concentration of BSA solution used in the modification process was 2 mg/mL. It took 30 min to modify BSA on the metal surface. The second chip was used in the nucleic acid detection. The flow cell was made of PDMS oligomer and a crosslinking agent (Sylgard 184). The mixture of PDMS and Sylgard 184 with a 10:1 ratio was degassed under vacuum, poured into a silanized glass mold, and then cured in an oven at 80 °C for 8 h.

### 2.3. Materials and Samples

The reagents used in the sensor performance test were deionized water and glycerol solution with a 20% volume fraction. Different concentrations of glycerol were obtained by diluting a glycerol solution of 20% volume fraction with deionized water.

In the nucleic acid hybridization experiment, phosphate buffer salt (pH 7.4 PBS 10×, diluted by deionized water 10 times) was the buffer solution. N-hydroxysuccinimide (NHS) and N-(3-dimethylaminopropyl)-N′-ethylcarbodiimide hydrochloride (EDC) were the activation solution. Ethanolamine was the closed solution, and 10 mM glycine-HCL was the regeneration solution. All the above solutions were purchased from GE Healthcare. Streptavidin (SA), DNA probes molecule (5′ - TTTCTACCCTTTGGTGCTAATGCCCATACT - 3′, 5′end with biotin), DNA (5′ - AGTATGGGCATTAGCACCAAAGGGTAGAAA - 3′) and non-complementary DNA sequence (5′ - TTTCTACCCTTTGGTGCTAATGCCCATACT - 3′) were kind gifts from the Institute of Biophysics, the Chinese Academy of Sciences.

### 2.4. Sensor Performance Test

After the SPR sensor was built, the angle scanning test was implemented with deionized water to test the practicability of angle scanning. During the test, a limit switch was used to return the angle of incident light to the origin that was the position of small angle. Then the angle was scanned from 44.438° to 59.849°. The scanning speed was 1.1607 × 10^−2^ m/s. The intensity signal of the reflected light was collected during the angle scanning. The signal acquisition frequency was 4.61 Hz.

The refractive index resolution of the instrument was measured by means of intensity modulation. No.0–No.10 glycerol was prepared and the volume fraction interval was 1%. No.0 was deionized water, No.1 was 1% glycerol, No.2 was 2% glycerol, and so on.

### 2.5. Experimental Protocol

**Figure 3:**
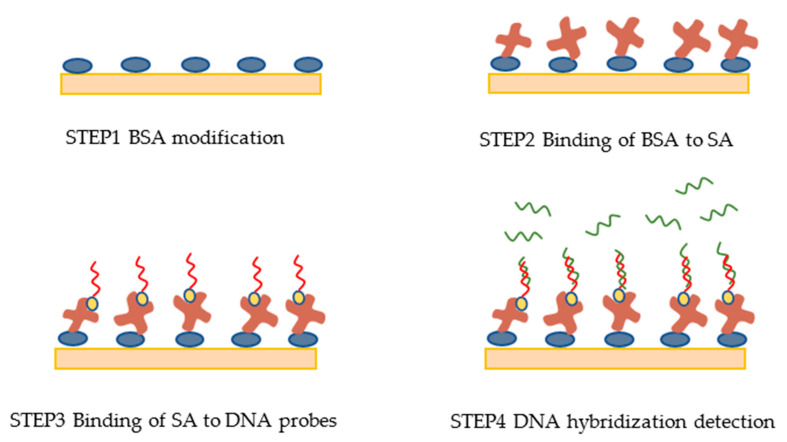
Schematic illustration of the experimental protocol.

There were four steps in the DNA detection experiment shown in Figure 3.

In step 1, a gold film chip was modified with BSA. Then, the modified chip and the flow cell were installed on the triple prism.

In step 2, PBS buffer solution was injected into the flow cell for 10 min to clean the surface of the chip. Then, the BSA molecules on the chip surface were activated by injecting the activation solution for 8 min. PBS buffer solution was then injected for another 6 min. Next, SA diluted 25 times with PH4.5 sodium acetate was injected into the flow cell for 10 min. SA was bound to the activated BSA. After that, PBS buffer solution was injected for 6 min to remove the free SA molecules.

To optimize the fixed quantity of SA molecule, SA diluted 10 times with PH4.5 sodium acetate was injected into the flow cell for 5 min. PBS buffer solution was then injected for 6 min to wash the surface, and ethanolamine was injected for 8 min to block the unbounded activation sites. This could prevent nonspecific binding in subsequent experiment.

In step 3, PBS buffer solution was injected for 5 min, followed by a solution of DNA probes (50 nmol/mL) for 5 min in order to bind the DNA probes to SA. PBS buffer solution was then injected for 6 min to wash the free DNA probes molecules. 

To optimize the fixed number of DNA probes, a solution of DNA probes (1 μmol/mL) was injected for 7 min.

Finally, in step 4, DNA detection hybridization experiment was implemented by injecting 0.01, 0.025, 0.05, 0.1, 0.25, 0.5 and 1 μmol/mL of DNA solution successively. After each test of a concentration of DNA solution, 10 mM glycine-HCL was injected for regeneration.

## 3. Results and Discussion

### 3.1. Angle Scanning Test

**Figure 4:**
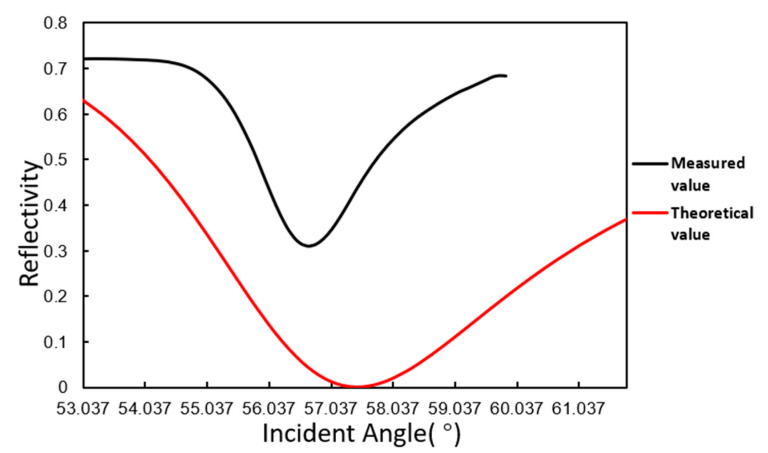
Angle scanning curve of deionized water.

The angle scanning test of the SPR sensor developed in this paper was performed. The measured value of deionized water was compared with the theoretical value of the SPR angle. The comparison result is shown in Figure 4. In the figure, part of the scan curve is only intercepted. 

The test result shows that the variation trend of light intensity is consistent with the theoretical value, and the SPR resonance angle is very close to the theoretical value, roughly 57°.

### 3.2. Refractive Index Resolution Test

#### 3.2.1. Unmodified Gold Film Chip

**Figure 5:**
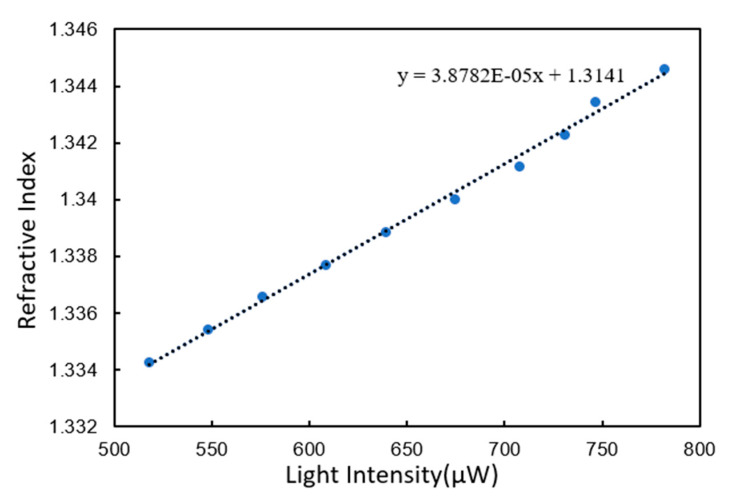
Linear regression curves of unmodified gold film chip.

The test was performed on an unmodified gold film chip. One hundred light intensity measurement data of glycerol at each concentration were selected to calculate the average light intensity. The calculated average light intensity was used as the abscissa, and the refractive index corresponding to each concentration of glycerol was used as the ordinate. Ten coordinate points can be obtained from No.1–No.10 glycerol. The linear regression curve of these 10 coordinate points is shown in Figure 5.

The curve was linearly fitted with linear correlation coefficient 0.996. The slope of fitting curve was 3.8782 × 10^−5^, which means that every 1 μW corresponded to 3.8782 × 10^−5^ RIU. Because most biological experiments measure near the SPR value of deionized water, deionized water was tested to obtain the noise of the instrument. A series of 500 measurements of deionized water were selected. The variance of the 500 points was 3.1979 × 10^−2^, which was used as the noise of the instrument. The refractive index resolution was calculated to be 3.72 × 10^−6^ RIU by multiplying sensitivity and noise three times.

#### 3.2.2. Gold Film Chip Modified by BSA

**Figure 6:**
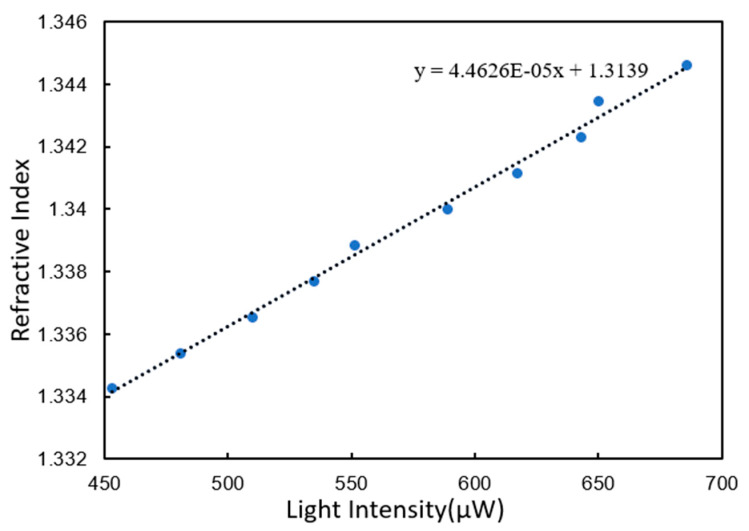
Linear regression curves of gold film chip modified with bovine serum albumin (BSA).

Since the nucleic acid detection process used the chip modified with BSA, the resolution of the chip modified with BSA was also tested. The test samples were still glycerol solutions with different concentration gradients. Ten coordinate points can be obtained from No.1–No.10 glycerol. The linear regression curve of these 10 coordinate points is shown in Figure 6.

The curve was linearly fitted with linear correlation coefficient 0.994. The slope of the fitting curve was 4.4626 × 10^−5^. Similarly, the noise was calculated using 500 measurements of deionized water. The variance of the 500 points was 1.4382 × 10^−2^. Similarly, the refractive index resolution was calculated to be 1.9254 × 10^−6^ RIU.

The results of refractive index resolution test show that the light intensity signal was positively correlated with the refractive index of the reagent. The SPR sensor developed in this paper was sensitive to the change in refractive index of the chip surface reagent. Its sensitivity could reach 3.72 × 10^−6^ RIU. Most of the surface of the chip modified with BSA had been occupied by BSA molecules. This made the reflected light intensity of the unmodified gold film chip change even more at the same glycerol concentration interval. When the glycerol solution was injected, the glycerol molecules on the unmodified gold film chip were closer to the metal surface and closer to the area where the surface plasmon wave dissipating field was strong. Because the y-coordinate parameters of the two tests were the same, the slope of the curve fitted by the refractive index resolution test of unmodified gold film chip was higher.

In order to avoid the influence of unstable light source, the noise measurement of the two chip surfaces was performed after the concentration gradient glycerol test. Compared with the chip modified with BSA, the non-specific adsorption was larger on the unmodified gold film chip so as to have a higher noise with it. 

### 3.3. DNA Probes Molecule Fixation

**Figure 7:**
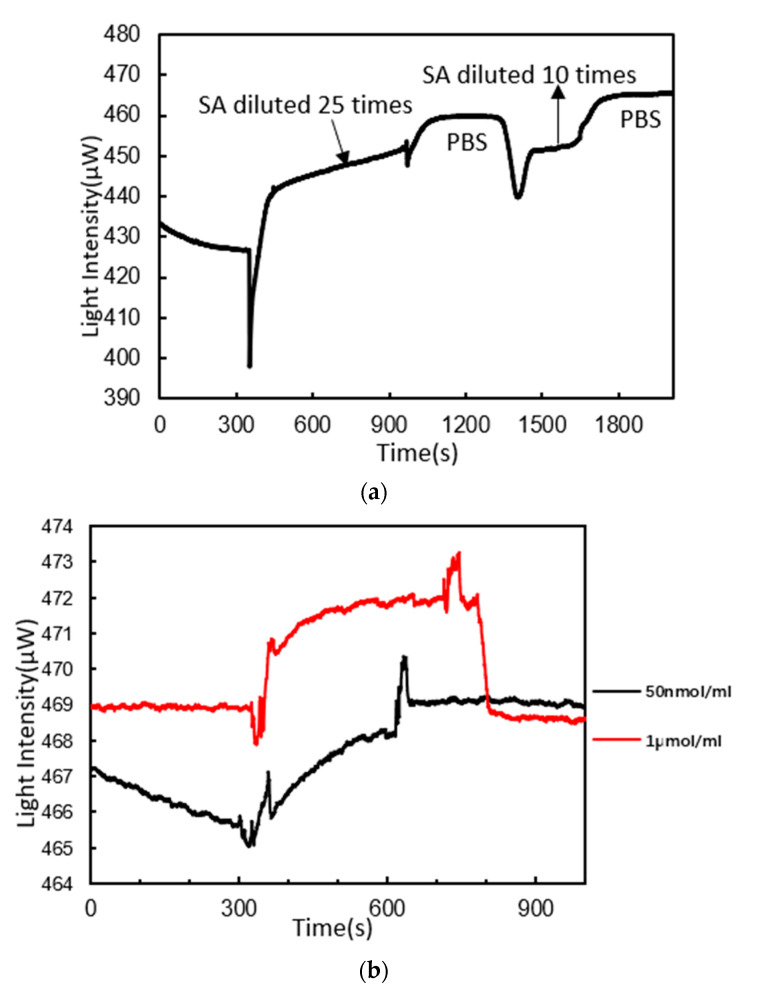
(**a**) Reaction curve of streptavidin (SA) binding to BSA; (**b**) reaction curves of DNA probes binding to SA.

The fixation of DNA probes was the key operation of this experiment to accurately detect nucleic acid. The first step in the fixation of DNA probes was to perform the binding of SA to BSA on the chip surface. The reaction curve is illustrated in Figure 7a. First, SA diluted 25 times with PH4.5 sodium acetate was injected. The light intensity curve started to go up gradually. Ten minutes later, the curve still showed an upward trend. PBS buffer solution was then injected for 6 min. The curve did not decline significantly. This indicated that SA had been bound to BSA. The curve increased by 33 μW after the reaction. SA diluted 10 times with PH4.5 sodium acetate was then injected. The curve increased by only 5 μW. The increase was not as obvious as before. This indicated that the fixed quantity of SA molecule was close to saturation.

The second step was to fix probe sequences of 5′- TTTCTACCCTTTGGTGCTAATGCCCATACT - 3′ (5′end modified with the biotion) on SA by the specific binding properties of SA to biotin. The reaction curve is illustrated in Figure 7b. A solution of DNA probes (50 nmol/mL) was firstly injected. The curve showed an exponential rising trend. Five minutes later, the curve tended to be stable. PBS buffer solution was then injected for another 6 min. The curve did not decrease significantly. The curve increased by 4 μW. A solution of DNA probes (1 μmol/mL) was then injected. The curve rose rapidly and then stabilized. After PBS buffer solution was injected, the curve decreased to the level before a solution of DNA probes (1 μmol/mL) injection. This indicated that the binding sites of SA to biotin were almost saturated and fewer probes could be bound to SA again.

### 3.4. DNA Hybridization Detection

**Figure 8:**
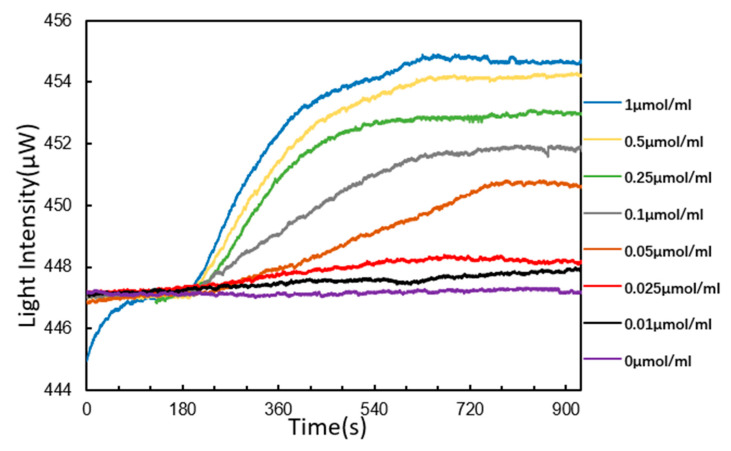
Reaction curves of DNA hybridization detection.

**Figure 9:**
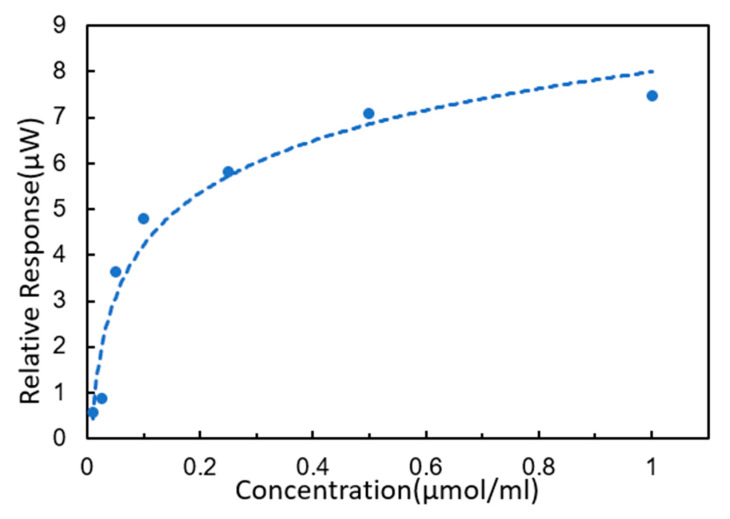
Standard curve of target DNA sequences.

According to the principle of complementary base pairing, single-strand DNA would be detected by DNA probes fixed on the chip surface. The sequence to be detected was 5′-AGTATGGGCATTAGCACCAAAGGGTAGAAA-3′. The reaction curves of DNA hybridization are shown in Figure 8. We started by injecting PBS buffer solution continuously for 3 min, and then started to inject the target solution after the curve was stable. When the target solution was injected, the curve showed an upward trend. The higher the concentration was, the faster the curve went up. The rising speed of curve of target solution (1 μmol/mL) was the fastest and the curve of the target solution (0 μmol/mL) remained stable all the time. In particular, the curves of the target solution at 0.25, 0.5 and 1 μmol/mL showed an exponential rise. The target solution was continuously injected for 9 min, and then all curves tended to be stable. After PBS buffer solution was injected for 3 min, these curves did not decline significantly. This indicates that the target DNA hybridized with the detection probes. Moreover, the higher the concentration was, the higher the reaction signal was, indicating that more target sequences were bound to the DNA probes fixed on the chip surface. Obviously, it showed that the SPR sensor in this paper was sensitive to changes in target solution concentrations from 0.01 to 1 μmol/mL. The response standard curve of the target sequence solution is shown in Figure 9.

In this experiment, the fixation of DNA probes was mainly divided into two steps. The first was to bind SA to the BSA on the chip surface. The second step was to fix the DNA probes sequence on the SA molecules. Although this method was complicated, it could make the probe molecules stand upright on the surface of the gold film, which was more conducive to hybridization. Moreover, due to the molecule size of BSA and SA, the probe hybridization sites were further from the chip surface. This effectively reduced the steric hindrance between the target sequence and the gold film, which also facilitated the hybridization reaction [35].

Response sensitivity is a key index of the performance of portable SPR sensor. The response sensitivity of an SPR sensor (i.e., the smallest change in the refractive index which can be measured by the sensor) can reach the order of 10^−7^ RIU for table-top laboratory systems. The response sensitivity of portable SPR sensors is of the order of 10^−6^ RIU [34]. Therefore, the sensitivity of the portable SPR sensor to DNA hybridization still needs to be improved. The improvement of sensitivity can be accomplished through two aspects. One is to improve the sensitivity of the light intensity acquisition device and the other is to improve the surface biochemistry reaction, making the DNA hybridization effect more obvious.

## 4. Conclusions

In this paper, by combining it with the classical Kretschmann prism structure, a portable multi-angle scanning SPR sensor with a simple angle scanning method and symmetrical optical path is developed for nucleic acid hybridization detection. The sensor is of high precision, simple control, and wide range of angle modulation. In addition, the resolution of angle scanning can reach 0.002°. In addition, the mechanical structure of the sensor is simple, stable and miniaturized, which is easy to carry and install. More importantly, it can greatly reduce the cost and facilitate mass production, thus accelerating the popularization and application of SPR sensor devices.

The sensor was used to implement sensitivity tests and hybridization experiments between DNA solutions in different concentrations and DNA probes. The results show that the SPR sensor has a high sensitivity of 3.72 × 10^−6^ RIU. The sensor has a fast response speed, and can successfully accomplish the hybridization detection of DNA with a solution of 0.01 μmol/mL.
